# Complete Genome Sequence of *Salmonella enterica* Serovar Enteritidis Bacteriophage f18SE, Isolated in Chile

**DOI:** 10.1128/genomeA.00600-15

**Published:** 2015-10-08

**Authors:** Cristopher Segovia, Ignacio Vasquez, Vinicius Maracaja-Coutinho, James Robeson, Javier Santander

**Affiliations:** a Microbial Pathogenesis and Vaccinology Laboratory, Faculty of Sciences, Universidad Mayor, Huechuraba, Chile; b Integrative Genomics PhD program, Faculty of Sciences, Universidad Mayor, Huechuraba, Chile; c School of Biotechnology, Universidad Mayor, Huechuraba, Chile; d Laboratory of Integrative Bioinformatics, Center for Genomics and Bioinformatics, Faculty of Sciences, Universidad Mayor, Huechuraba, Chile; e Instituto Vandique, João Pessoa, Brazil; f Microbiology Laboratory, Institute of Biology, Pontificia Universidad Catolica de Valparaiso, Valparaíso, Chile; g School of Life Sciences, Arizona State University, Tempe, Arizona, USA

## Abstract

Bacteriophage f18SE was isolated from poultry sewage in Olmue, Chile, and lytic activity was demonstrated against *Salmonella enterica* serovar Enteritidis and serovar Pullorum strains. This bacteriophage has a 41,868-bp double-stranded DNA (ds-DNA) genome encoding 53 coding sequences (CDSs) and belongs to the family *Siphoviridae*, subfamily *Jerseyvirinae*.

## GENOME ANNOUNCEMENT

*Salmonella enterica* serovar Enteritidis is one of the most common causes of salmonellosis worldwide. *S*. Enteritidis is typically transmitted by food-products derivate from the poultry industry. Since phage prophylaxis can reduce the use of antibiotics, we isolated the bacteriophage f18SE from poultry sewage (1). This phage has a broad host range infecting *S*. Enteritidis PTs, *Salmonella* Pullorum, and *Salmonella* Typhimurium serovars (1). The morphology of the viral particles is similar to that of phage λ. It has an icosahedral head of 30 × 24 nm, a tail of 95 × 5 nm, and basal spikes. Although, the genome annotation included fibers, they could not be visualized by transmission electron microscopy. f18SE consists of double stranded DNA with a characteristic *EcoR*I digestion profile (1). According to its characteristics, the phage f18SE belongs to the family *Siphoviridae*, subfamily *Jerseyvirinae* (2). f18SE has been successfully evaluated as prophylactic agent in *Caenorhabditis elegans* (3) and chicks (4). Its stability under harsh conditions (pH and T) and on inoculated eggs is significantly high (5). Also, its membrane attachment molecule is the oligo-polysaccharide of the lipopolysaccharide (6). The potential use of phage f18SE in typification, vector development, and biocontrol strategies can be anticipated, and thus the sequence of this phage can contribute to its potential use and to *Salmonella* phage biology.

f18SE DNA was purified according to reference (7). DNA sequencing was performed using the next generation sequencer (NGS) Illumina MiSeq (8× coverage) at Universidad Mayor, Center for Genomics and Bioinformatics (Huechuraba, Chile). The sequences were assembled using CLC Genomics Workbench 8.0.1, resulting in a unique contig. f18SE contains 41,868 bp and has a G+C content of 49.8%. The potential coding sequences (CDSs) were initially annotated using BLASTn. The predicted proteins were analyzed using BLASTp. The genome contains 53 predicted genes, with an average gene length of about 875 bp. Seventeen of the genes are rightward oriented, while thirty-six are leftward oriented. Forty-three coding sequences begin with the start codon AUG, while ten begin with the start codon AUU. Based on the predictions, this phage genome contains genes for phage replication, structure, and lysis. Open reading frames (ORFs) were found for putative homing endonuclease, helicase, and DNA polymerase. The ORFs for terminase, head morphogenesis protein, putative tail protein, and tail fiber protein were found. No lysogenization genes, such as site-specific integrases and repressors, were identified. The ORFs for holin and endolysin were also found. Alignment and molecular phylogenetic analysis by maximum likelihood method (8–10) shown that three phages closely related to f18SE are *Salmonella* phages L13 (GenBank accession no. KC832325), wksI3 (GenBank accession no. JX202565), and SS3e (GenBank accession no. AY730274). 

### Nucleotide sequence accession number.

The complete genome of the *Salmonella* Enteritidis f18SE has been deposited in GenBank under the accession no. KR270151.
